# A simplified model of biosonar echoes from foliage and the properties of natural foliages

**DOI:** 10.1371/journal.pone.0189824

**Published:** 2017-12-14

**Authors:** Chen Ming, Hongxiao Zhu, Rolf Müller

**Affiliations:** 1 Department of Mechanical Engineering, Virginia Tech, Blacksburg, Virginia, United States of America; 2 Department of Statistics, Virginia Tech, Blacksburg, Virginia, United States of America; 3 Shandong University - Virginia Tech International Laboratory, Shandong University, Jinan, China; University of Western Ontario, CANADA

## Abstract

Foliage echoes could play an important role in the sensory ecology of echolocating bats, but many aspects of their sensory information content remain to be explored. A realistic numerical model for these echoes could support the development of hypotheses for the relationship between foliage properties and echo parameters. In prior work by the authors, a simple foliage model based on circular disks distributed uniformly in space has been developed. In the current work, three key simplifications used in this model have been examined: (i) representing leaves as circular disks, (ii) neglecting shading effects between leaves, and (iii) the uniform spatial distribution of the leaves. The target strengths of individual leaves and shading between them have been examined in physical experiments, whereas the impact of the spatial leaf distribution has been studied by modifying the numerical model to include leaf distributions according to a biomimetic model for natural branching patterns (L-systems). Leaf samples from a single species (leatherleaf arrowwood) were found to match the relationship between size and target strength of the disk model fairly well, albeit with a large variability part of which could be due to unaccounted geometrical features of the leaves. Shading between leaf-sized disks did occur for distances below 50 cm and could hence impact the echoes. Echoes generated with L-system models in two distinct tree species (ginkgo and pine) showed consistently more temporal inhomogeneity in the envelope amplitudes than a reference with uniform distribution. However, these differences were small compared to effects found in response to changes in the relative orientation of simulated sonar beam and foliage. These findings support the utility of the uniform leaf distribution model and suggest that bats could use temporal inhomogeneities in the echoes to make inferences regarding the relative positioning of their sonar and a foliage.

## Introduction

Many echolocating bat species can navigate [1–3] in dense vegetation and find insect prey or other foods such as nectar [4] and fruit [5] among foliage based on information obtained from ultrasonic biosonar echoes [6]. Hence, the nature of foliage echoes is likely to play a critical role in the function of the biosonar system of bats that occupy such sensori-ecological niches. Foliage echoes could serve as sources of information for a variety of sensory tasks: For example, the ability to distinguish different foliage types based on their echoes could support habitat selection or the identification of landmarks. The ability to estimate the average orientation of leaves in a foliage could allow bats to obtain information on the orientation of a foliage surface and hence support following a contour along a vegetation edge. Natural foliages are highly complex (bio)sonar targets that consist of many ultrasound-reflecting facets (esp. leaves). As a consequence, foliage echoes are superpositions of many (tens, hundreds, or even thousands) individual reflections. Because it is not possible in practice to know the position and orientation of all facets that contribute to given foliage echoes, such echoes have to be treated as random processes. It has been demonstrated that bats can distinguish signals with different roughness [7] which indicates that the animals have the ability to distinguish between echoes that are realizations of random processes based on differences in certain statistical invariants. On the computational side, a few studies have demonstrated that the classification of foliages based on echoes is possible [3, 8–10]. These classification studies have typically investigated distinct foliages or foliage categories. Hence, they shed little light on the limits for making finer distinctions between foliages or the ability to estimate continuous foliage parameters (e.g., leaf density or leaf size). Simulation studies based on computational foliage models hold promise for gaining insight into these topics because they allow it to vary foliage parameters continuously or in very small steps where many realizations for each step can be created and the ground truth is always known. A condition for this approach to succeed is that the computational foliage models are able to capture all salient foliage properties accurately. Yovel et al. [9] have presented a foliage model that treats leaves as point reflectors, i.e., each leaf has an omnidirectional beampattern. The model also tested two different leaf distributions (uniform Poisson and leaf clusters placed with a Poisson model). The results showed that cluster-distribution model provided a better fit to measured echo power spectral densities than the uniform-distribution model. In prior work by the authors, a foliage model includes leaf size and leaf orientation as additional parameters [11]. Given that leaves are likely to fall into the Mie scattering region [12] of the bat biosonar pulses, i.e., the leaves are on a similar size scale as the wavelengths used by the bats, these two parameters can be expected to have a strong impact on the echo contributions of individual leaves. The model developed by the authors predicts that the echoes of the entire foliage are also impacted by those parameters and hence could provide an opportunity to estimate average leaf size and orientation from the echoes. For the insights gained through computational foliage models to be valid for actual foliage echoes and hence the sensory ecology of echolocating bats, it is important that the simplifying assumptions of the model are close enough to reality to replicate the key properties of the echo-generating process accurately. Hence, the goal of the work presented here has been to look into two key features of the model, the disk-shaped leaves and their homogeneous distribution in space. To compare the impact of leaf geometry and size on the scattering properties of the leaves, sample leaves from different plant species were ensonified and their impulse responses were recorded experimentally. As part of these experiments, the extent of shading between leaves was also assessed. To assess whether the uniform leaf distribution model needs to be improved in the light the inhomogeneities that can be found in real tree foliages, especially due to branching patterns and the distribution of leaves along the branches, the foliage model was modified so that the leaves were placed using an established model for tree branching patterns (Lindenmayer or L-systems). Along with this next generation of the foliage model, a measure for echo inhomogeneities has been developed and applied to quantify the impact of foliage inhomogeneity as well as the orientation of the sonar with respect to a foliage.

## Methods

### Model

**Fig 1:**
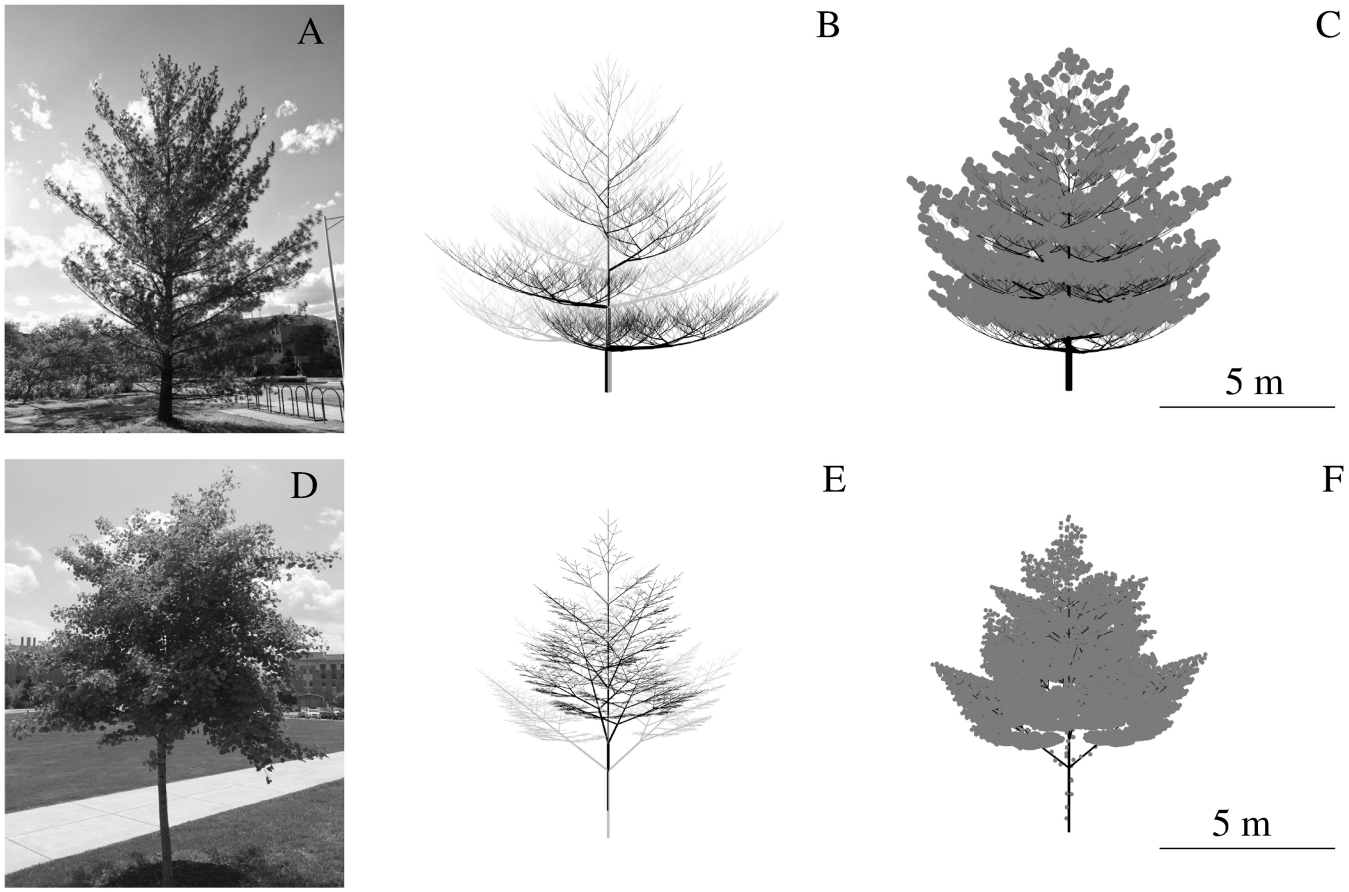
Tree specimens with their respective digital tree models constructed using L-systems. A) Eastern white pine (*Pinus strobus*); B) L system model of the same species [15] with its 180° rotated version superpositioned to increase the branch density; C) same as B) with leaves added. D),E) and F): same with A), B) and C) for a young ginkgo (*Ginkgo biloba*) except for 90°’s rotation and that the rotated version is lifted half length of the initial branch along *z* axis.

The computational foliage model studied here simplifies the geometry of the leaves in a foliage as circular discs. The exact far-field solution for the beampattern of an acoustically hard disc can be expressed as a sum over an infinite series composed of spheroidal wave functions. A highly accurate numerical evaluation of the exact solution is possible truncating the series based on a threshold criterion [13]. However, since this approach is computationally expensive, a cosine function was used to approximate the amplitude of the scattered field as a function of leaf size, sound wavelength, and incident angle [11]. In the current work, the positions of leaves in the model foliage were determined in two alternative ways: In the first alternative, the leaves were uniformly distributed in a rectangular box in front of sonar as has been reported previously [11]. In the second alternative, the leaf positions were determined based on a biomimetic branching pattern obtained from a Lindenmayer (L)-system [14] combined with ad-hoc rules for how leaves grow around each branch. For the uniform distribution, the initial leaf domain was a rectangular box with walls placed so that they enclosed the entire volume where the spreading losses associated with two-way transmission between sonar and each leaf did not exceed 80 dB. For a -3 dB beamwidth of sonar to 30°, this approach resulted a leaf domain with dimensions 9×4×4 m, where all edge lengths were rounded up to the nearest integer. The leaf domain was positioned one meter away from sonar to conform to the far-field assumption made in the echo simulation. The coordinates of each disc were drawn from a uniform random distribution that covered the entire rectangular box. The two-way spreading loss associated with each leaf position created within the rectangular box were tested against the threshold of 80 dB. Leaves in positions with spreading losses that exceed this threshold were discarded, since their contributions to the echo were too small to warrant the computational echoes. Lindenmayer (L-) systems [14] were used to create the inhomogeneous leaf distributions tested here. L-systems are recursive algorithms for creating branching patterns that mimic the growth of biological trees. Each recursion creates an additional level of growth/branching. Two sets of L-system parameters were evaluated in order to create models for two different tree species, eastern white pine and ginkgo. An adult eastern white pine (*Pinus strobus*, Fig 1A) was mimicked by an L-system tree model of growth level 13 (Fig 1B, gray). The tree model fit into a volume of 2016 m^3^ (width, depth, and height: 12, 14, and 12 m). The pine model was based on a bifurcation-type L-system, i.e., two child branches were generated from the tip of each mother branch. Child branches and their mother branches defined a plane that was perpendicular to the plane determined by the mother branch and the branch two growth levels prior to the current branch. The branching angle defines how much the directions of child branches deviate from the direction of their respective mother branch. Since the branching pattern of eastern white pine is monopodial, i.e., there is a single main axis (trunk) to which lateral branches remain subordinate, one of the two branching angles in each iteration was zero. The other branching angle started at 90° at the first level and decreased by about 5.8° in every higher level until it reached 20° at the 13th level [15] (Fig 1B, gray). In each iteration, the length of the child branches became shorter by a constant contraction ratio, which was set to 0.9 for stem branches and 0.7 for side branches. Inspired by real pine trees, thirty 15-cm-long bundles of needles placed spirally around a 4.5-cm-long part of each terminal branch starting from the tip. The branching angle of those bundles was set to 75°. Each bundle was then replaced by five discs placed randomly in a cube with an edge length of 1 cm, the center of which coincided with the tip of the bundle. The orientations of the five discs were defined by vectors between the origin of the bundle and points picked from a uniformly random distribution within the cube. The radial direction of disks were aligned with this vector. The radius lengths of all discs in pine tree were drawn from a Gaussian distribution with a mean of 2 mm and a standard deviation 0.2 mm. For the ginkgo (*Ginkgo biloba*, Fig 1D) model, the maximal growth level was set to eight resulting in a volume of 490 m^3^ (width, depth, and height: 7×7×10 m, Fig 1E, gray). Ginkgo trees have a ternary branching pattern, where three child branches are generated in each branching process: one in the direction of the mother branch, the other two branching off to opposite sides with branching angle of 50°. The contraction ratios were 0.83 for the central branch and 0.62 for each of the two side branches [15]. Four evenly spaced leaf nodes, i.e., short shoots that bear the leaves, were placed on each terminal branch. The leaf nodes divided the terminal branch into four equal parts with one node occupying the tip. Leaf nodes were placed on the non-terminal branches in a similar fashion (even spacing along two opposite sides of the branch, 4 leaves at each node). Leaf nodes placed at the tip of a terminal branch were oriented in the same direction as the terminal branch; all other leaf nodes were oriented perpendicular to the branch they were placed on. The orientation of first node to be generated was drawn from a uniform random distribution of all orientations perpendicular to the branch. All other nodes were then placed by rotating the orientation of the preceding node by 90°. The center positions for the leaves belonging to a leaf node on the tip of a terminal branch were selected from a uniform random distribution over a rectangular box (10×10×20 cm) centered on the node. Leaves from in-branch nodes were displaced from their respective node by a random vector (with all coordinates drawn independently from a uniform distribution) that was added to the vector specifying the direction of the node with a weight of one tenth. The radii of the leaves were drawn from a Gaussian distribution with mean 2.5 cm and standard deviation 0.25 cm.

Since the tree models that were generated based on the rules described above appeared less dense than natural trees of the same species, a copy of each tree was made by rotating pine and ginkgo 180° and 90°, respectively. The two copies were then added together (Fig 1B and 1E, black). For ginkgo, the copy was also raised by half the length of the initial branch to insure that branches were spaced along the length of the stem.

The beampattern of the simulated sonar was represented by a product of two Gaussian functions, one in elevation and the other in azimuth. For the uniform model, the sonar was always aimed at the foliage. For the uniform leaf distributions as well as the L-system tree models, experiments with different relative positions and orientations between the biosonar beam and the tree were conducted. Specifically, the following three scenarios were investigated: (i) approach: the sonar was aimed at center of the tree while being translated over distances from 9.5 m down to 2.5 m towards the center of the tree, (ii) angular scan: the aiming direction of the sonar (-3 dB beamwidth 50°, constant target distance about 2 m) is rotated from facing the center of the tree (defined as 0°) to a maximum rotation angle of 90°, and (iii) beam widening: the sonar is kept facing at the center of the tree from a constant distance of about 2 m while the -3 dB two-sided beamwidth is varied between 1° and 60°. To perform matching experiments with the uniform leaf distribution model that can be compared to the results obtained from the L-system tree models, the following adjustments were made to the uniform model: the size of the cuboid leaf domain was adjusted to match the duration of the echoes from the L-system model trees and the uniform leaf density was selected so that the number of leaves in the beam matched those of the L-system model. The leaf size used in uniform-distribution models matched that in L-system models for the two species, respectively. The mean orientation angles for ginkgo and eastern white pine were 45° and 5° with standard deviation 5°, respecitively.

**Fig 2:**
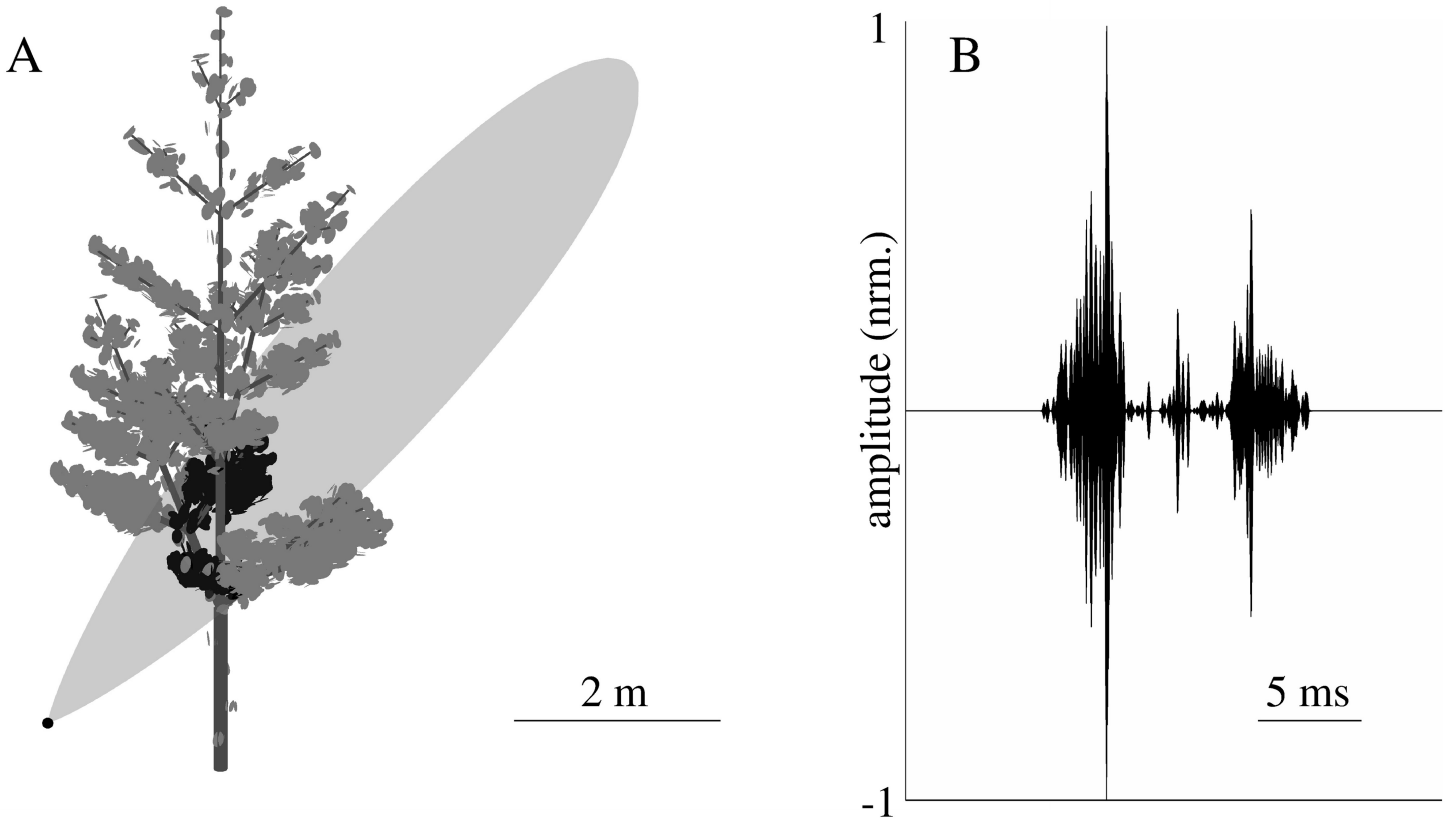
Example of a tree model created using an L-system. A) relative position of tree model and simulated sonar beam (sonar position indicated by black dot, -3 dB beamwidth 10°). The leaves with positions within the -3 dB beam contour are colored in black. B) Numerical prediction of impulse response corresponding to the situation depicted in A).

The numerical echo predictions (Fig 2B, resulted from scenario Fig 2A) for all studied foliage models and sonar-position scenarios were obtained for a frequency band between 60 and 80 kHz which is similar to the second (and strongest) harmonic in the biosonar pulses of the greater horseshoe bat (*Rhinolophus ferrumequinum* [16]). The echoes were simulated by predicting the transfer function for each leaf in this frequency band at 2000 evenly spaced frequencies and complex addition of the individual leaf echoes in the frequency domain. Time-domain signals were derived by performing an inverse Fourier transform (with a 2000-point Hanning window).

### Measure of inhomogeneity

To compare the echoes generated by the uniform leaf-distribution model and the L-system-based models, the hypothesis was investigated that an inhomogeneous distribution of the leaves could give rise to a temporal inhomogeneity in the echoes. To this end, a measure to quantify the inhomogeneity of the echo amplitudes over time was devised as follows: The envelope of each echo was calculated as the magnitude of an analytic signal obtained from the original waveform by virtue of the Hilbert transform [17]. The beginning and the end of the echo were determined by comparing the envelope amplitudes against a threshold that was set to 100 dB above the numerical noise level defined by the average envelope amplitude away from any simulated echoes. The starting time of the echo was taken to be the time of the first sample that exceeded the threshold and the end was the time of last sample to exceed the threshold. Due to the limited bandwidth of the echo envelopes, the sampling rate of the echo envelope was reduced to 50 kHz from the original 400 kHz that was used for the original echo waveforms. Depending on echo duration, this resulted sample numbers that ranged from a tens of samples up to three thousand samples. Each simulated echo envelope was padded with 50 zeros on each end to ensure that the length of even the shortest echoes was sufficient for the analysis. To gauge inhomogeneity in the echo envelope, the duration of each echo (including the padding) was divided into 100 time windows. A replica of the echo was then created where the time sequence of the 100 windows was randomly permuted. Finally, the root mean square difference between the average amplitudes of the original and the permuted envelopes across all 100 windows was computed as a measure of the temporal inhomogeneity in the echo envelope.

### Leaf measurements

Acoustic measurements of leaves were carried out in an anechoic chamber (inner dimensions: 5.4×4.1×2.4 m). In order to assess the impact of the variability in leaf geometry on target strength an experiment with 100 freshly cut leaves of the leatherleaf arrowwood (*Viburnum rhytidophyllum*) was conducted. For each of the 100 leaves, 50 echoes were measured while the leaf was positioned at a fixing distance of 1.5 m to the sonar. To assess variability across different plant species, the same measurements were carried out for another ten leaves, each from a different species (Table 1). The leaves in the latter experiment were pressed and dry when the measurements were undertaken. The area of each leaf tested was determined from a digital image and used to represent the leaf size by an “equivalent radius”, i.e., the radius of the circle with an area equal to the measured area of the leaf.

**Table 1:** Tree species with their respective estimated equivalent leaf radii used in the acoustic leaf characterizations.

Common name	Scientific name	Equivalent radius (cm)
Euonymus, deciduous	*Euonymus alatus*	1.7
Paperbark maple	*Acer griseum*	1.7
River birch	*Betula nigra*	1.8
Ornamental cherry	*Prunus spp*.	1.8
American beech	*Fagus grandifolia*	2.1
Higan cherry	*Prunus subhirtella*	2.5
Ginkgo	*Ginkgo biloba*	2.5
Redbud	*Cercis canadensis*	2.8
Swamp white oak	*Quercus bicolor*	4.6
Pin oak	*Quercus palustris*	5.0

To assess the impact of shading between leaves, two cardboard disks of radius 1.6 cm were used. The distance between the two discs was varied from 25 cm to 150 cm in increments of 25 cm. The following three measurements were repeated for each distance: First, the distant (“back”) disk was ensonified without the other (“front”) disk being present. Second, the front disk was placed right along the line of sight from the sonar to the back disk (“fully shaded”). Third, the front disk was displaced to the side by its radius to produce a condition of “partial shading”. In all these measurements, the position of the back disk remained fixed. The change of distance between two disks were realized by moving the first disk to a different position at each time. The impulse response from the two discs can be distinguished by time.

**Fig 3:**
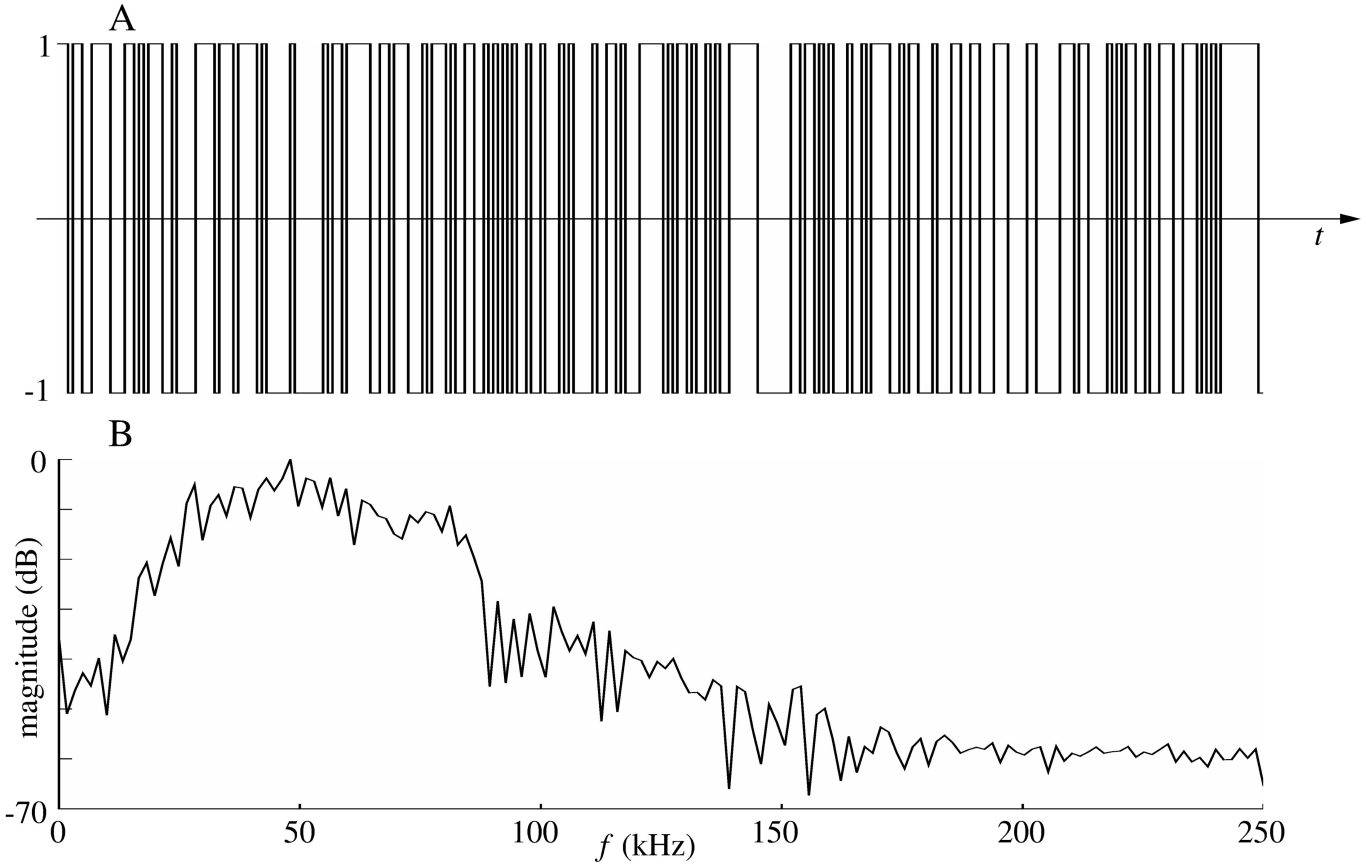
Acoustic signals used in the experiments. A) Example realization of an MLS sequence of length 255. B) Spectrum of an echo triggered by an MLS pulse that was aimed at a cardboard disc (10 cm diameter).

The impulse responses of leaves and disks were recorded with a biomimetic sonar composed of one electrostatic ultrasonic loudspeaker (Series 600 open face ultrasonic sensor with 4.3 cm diameter, SensComp, Inc., Livonia, MI USA) with a two-sided -6 dB beamwidth of 15°. The loudspeaker was driven by a power amplifier (AA-301HS, A.A. Lab Systems Ltd. Ramat-Gan, Israel). Echo signals were recorded by two MEMS capacitive microphones (SPU0410LR5H, Knowles Electronics, LLC. Itasca, IL USA) integrated on pre-amplifier boards (Momimic, Dodotronic, Rome, Italy). A single A/D&D/A conversion board (NI-6351, National Instruments Corp., Austin, TX, USA) was used to interface the setup with the control computer. Two conical horns (base radius 2.5 cm, top radius 0.25 cm, height 10 cm) were placed in front of microphones. The sonar head was mounted on a tripod which was adjusted to the same height at which the leaves and disks were mounted with thin filament (diameter about 0.3 mm, 12 lb fishing line). The pulse used in the experiments was a periodic pseudo-random sequence (maximum length sequence, MLS [18], see Fig 3). Each sequence consisted of 255 samples which results in a pulse length of 0.5 ms with the 500 kHz sampling frequency employed. The choice of sample number and sampling frequency was a compromise between the requirements that the signal should be short so that the echoes from close discs can be distinguished, and that the signal should have a lot samples to carry enough energy for a good signal to noise ratio.

The maximum value of the recorded impulse responses was determined by fitting a 2nd order polynomial to an 11-point window taken from the envelope of the impulse response in the vicinity of the maximum. The median across all the impulse responses maxima from the 50 echoes that were recorded for each target was used to represent the target’s acoustic strength. To compare the predictions from the disk model to the leaf data, a scalar scaling parameter was used to fit the predictions from the model to the measured data in a least-squares sense.

## Results

**Fig 4:**
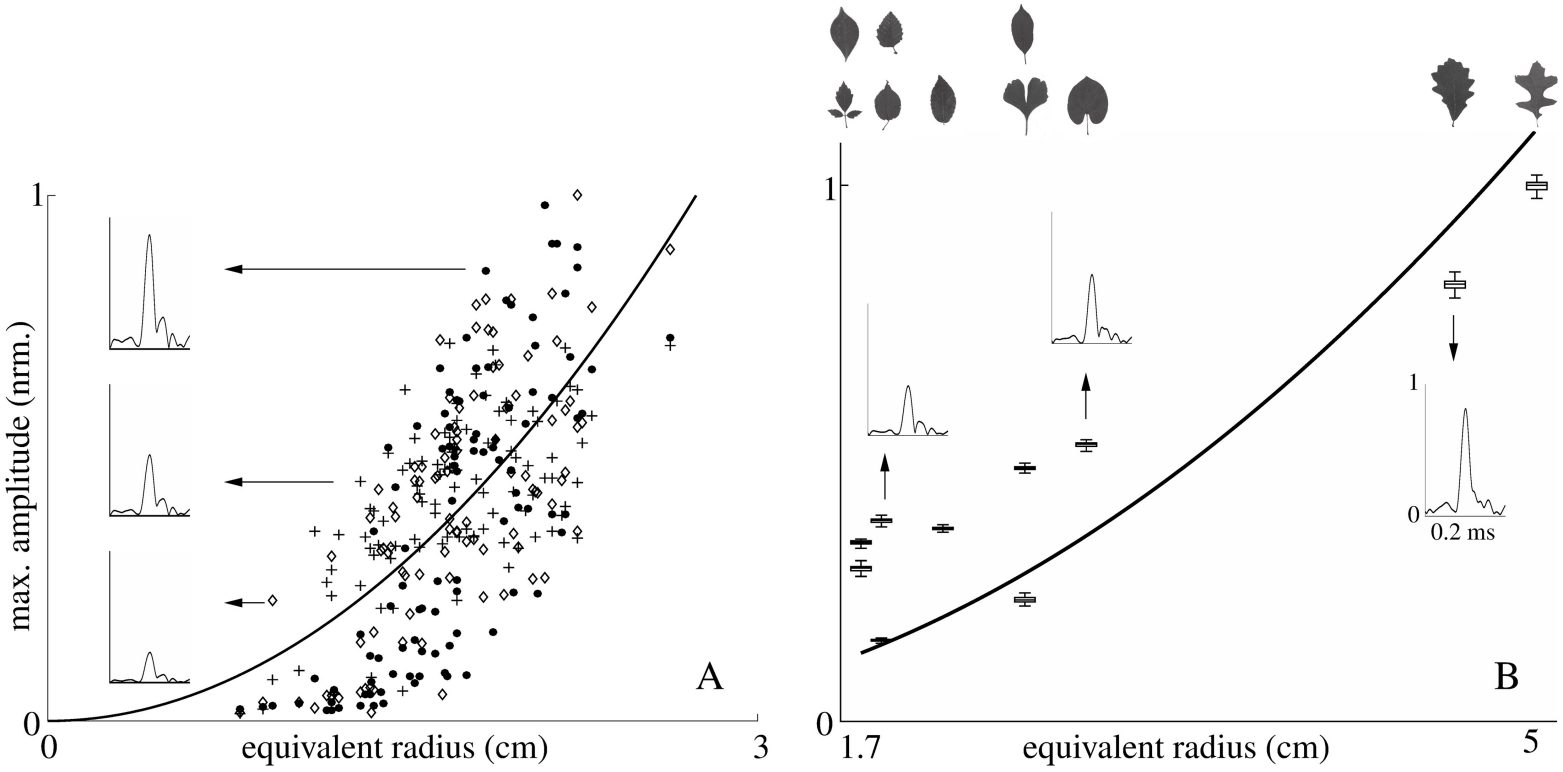
Leaf target strength as a function of equivalent radius. A) Measured values of leaf target strength (maximum impulse response amplitude) from 100 leaf samples of leatherleaf arrowwood (*Viburnum rhytidophyllum*) together with the prediction from the disk model (solid lines). The measurements were repeated three times and each repetition is indicated by a different marker: filled circle (first repetition); diamond (second repetition); plus sign (third repetition); simulation: solid line. 50 echoes were collected for each leaf in each measurement. Each symbol in the plot is the median of 50 impulse response maximums. The fit of the model was accomplished by picking the value of a scalar scaling factor that minimized the deviations between data and model in a least-square sense. B) Measured values of leaf target strength of broad leaves across 10 species are shown together with the predictions from the disc-model (solid line, model fitting as described in panel A). The silhouettes of the leaves measured are shown in the top of the respective data points. The measurement of ginkgo leaf was conducted with a different sampling frequency (250 kHz); all other conditions were identical. The insets in the panels describe envelopes of example impulse responses in the measurement of individual leaves.

Within the same tree species (leatherleaf arrowwood), the measured target strengths of the leaves showed a positive, monotonic trend to increase as a function of equivalent leaf radius (Fig 4A). This trend was in reasonably good agreement with the prediction from the disc-model although a large amount of scatter was observed in the individual data points (coefficient of determination 0.45).

A similar monotonic trend of target strength increasing with equivalent leaf radius was also found across leaves from different species (Fig 4B). The coefficient of determination was similar to the single-species case (0.58) and the data from the different species seems to fall well within the variation seen in the single-species data set.

**Fig 5:**
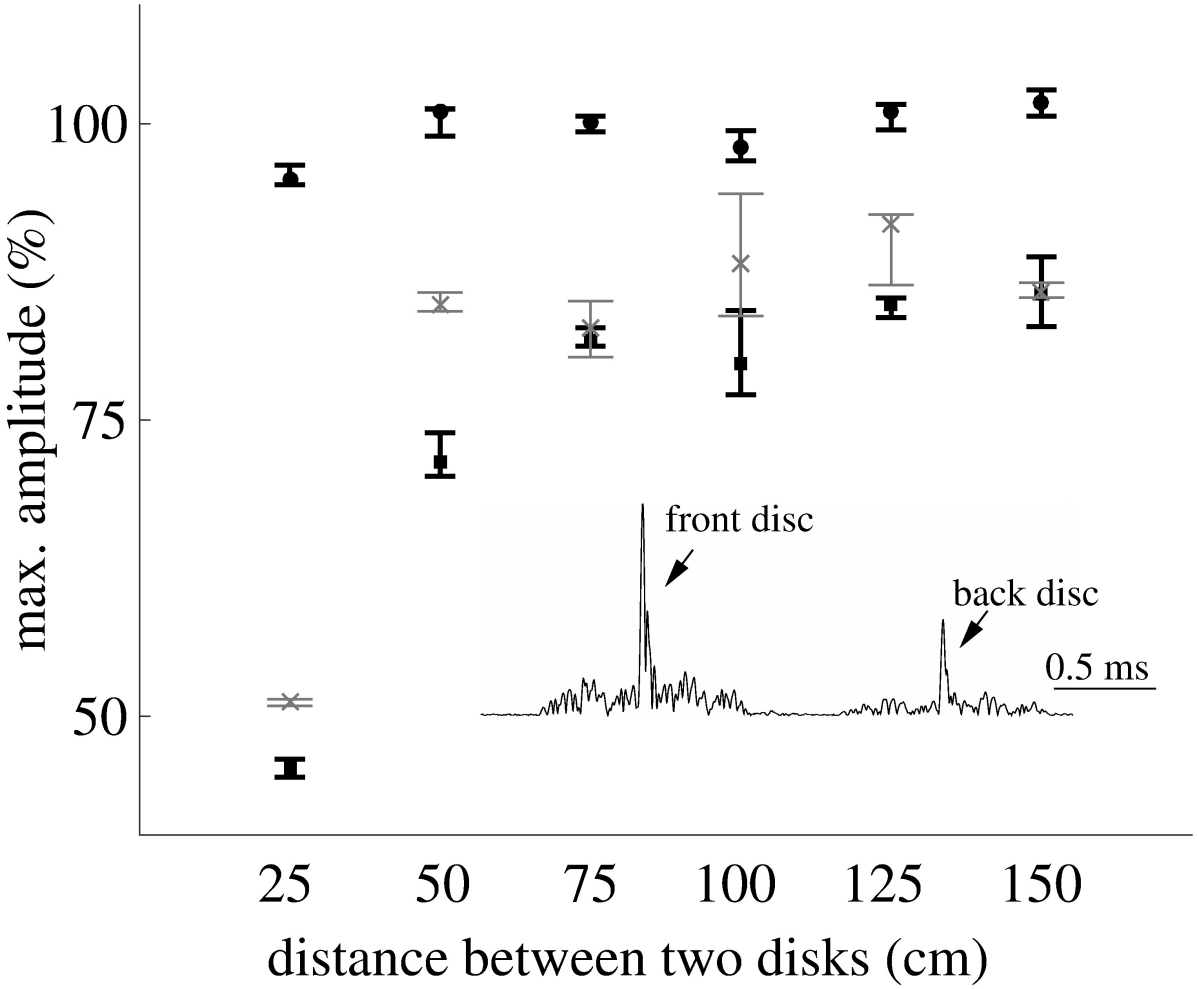
Experimental characterization of shading effects between leaves on target strength. Circles: no shading, crosses: complete shading, squares: partially shaded. The diameters of two disks were 3.6 cm. 50 echoes were collected for each situation, the markers represent the mean and the error bars represent the 75th and 25th percentile of the data set. All amplitude values were normalized with the mean of the impulse response maximums when there was no shading. The inset shows the envelopes of the impulse responses from two discs (the back disc was fully shaded) with a spacing of 25 cm, which indicates that the impulse response from each disc can be distinguished by time.

The acoustic measurements conducted on the disc pairs demonstrated the existence of acoustic interaction effects between leaves that depended on the distance between the discs (Fig 5). At the smallest distance surveyed (25 cm), shading resulted in a drop in target strength of slightly less than 50%, i.e., less than 6 dB. At larger distances, the shadowing effect quickly decreased to reach less than 25% (2.5 dB) at a distance of 75 cm. The differences between the fully shaded and partially shaded conditions were small in general. However, when the two discs were 25 cm or 50 cm apart, the impulse responses from the fully-shaded disc were stronger than that of the partially shaded disc at the same distance from sonar.

**Fig 6:**
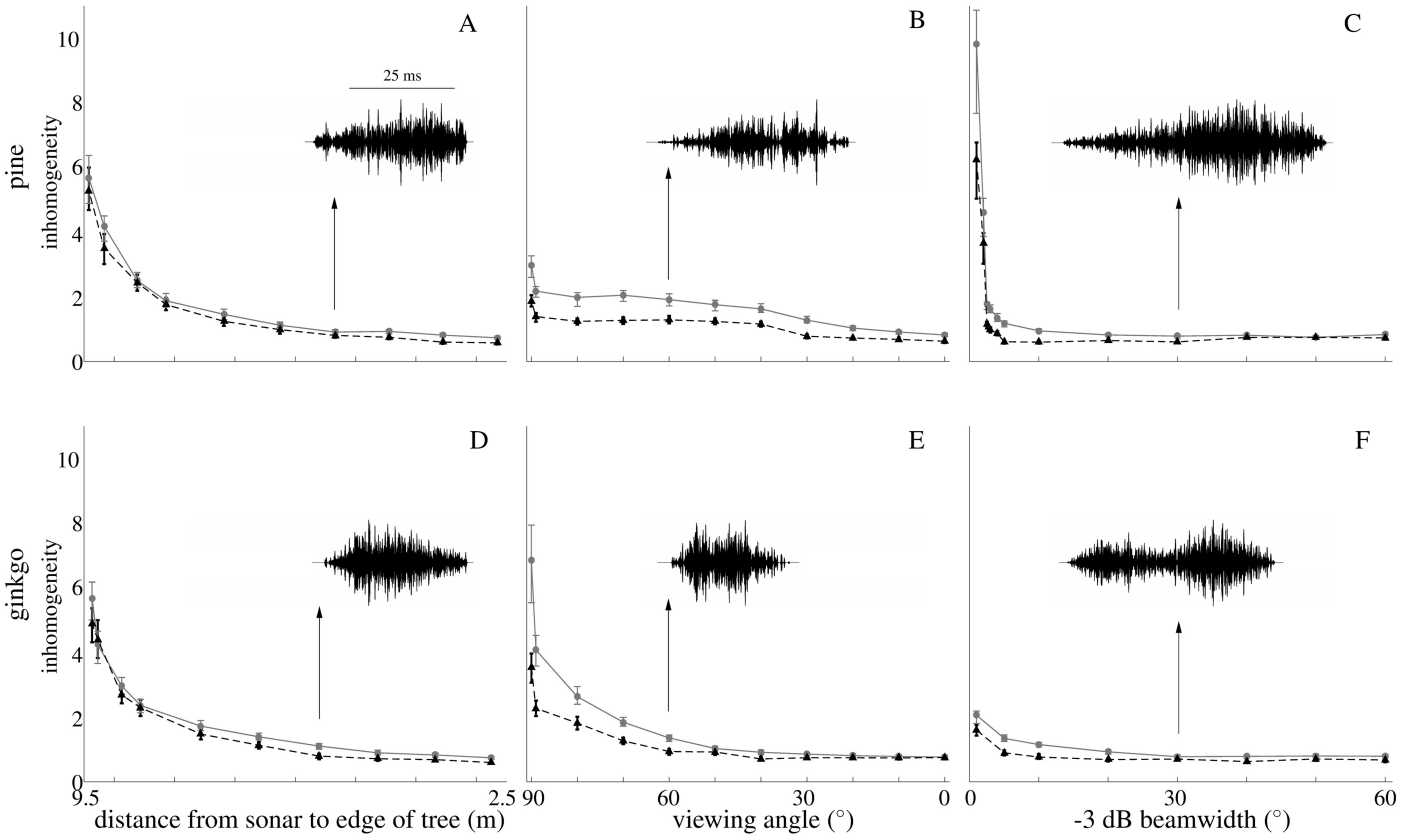
Echo envelope inhomogeneity for the L-system tree models compared to a uniform-distribution model reference. Solid lines: L-system tree models, dashed lines: uniform-distribution model. A) and D) Straight approach towards the foliage (A pine, B ginkgo); B) and E) Angular scan with viewing angles ranging from 90° to 0° (oriented straight at the center, B) pine, E) ginkgo); C) and F) Change in -3 dB beamwidth (C pine, F ginkgo). The leaf density of the uniformly distributed reference models was adjusted in each condition to match the number of leaves in the sonar beam of the two L-system models. In addition, the size of the leaf domain in the uniform leaf distribution model was adjusted to match the echo length in L-systems. Each point represents the mean of 100 experiments, the error bars indicate the minimum and maximum values in each data set. The insets in each panel show an example waveform with a parameter value in the middle of the range shown in the main figure. Inhomogeneity is the root mean square of the difference between the two means (mean of original envelope and mean of permuted envelope) across 100 windows.

The measure of temporal inhomogeneity used here produced non-zero values for all foliage models tested and all relative positions and orientations of the sonar (Fig 6). The values of the measure obtained for the uniform leaf distribution model were consistently lower than those that resulted from the two L-system based models for the same experimental condition. However, the differences between the two different model categories were much smaller than the changes that could be elicited by changing the relative position, orientation, or the width of the biosonar beam. Hence, the inhomogeneity measure was found to be far more sensitive to the position and orientation of the sonar relative to the foliage than to the inhomogeneity in the leaf distribution within the foliage.

The three experimental scenarios for changing the sonar position, orientation, and beamwidth in the simulations were each found to have a substantial impact on the temporal inhomogeneity of the echoes (Fig 6). As the sonar approached the model foliage, the inhomogeneity decreased monotonically by a factor of approximately six for all three foliage models (Fig 6A and 6D). For the angular sonar scan, the ratio between the highest and lowest values differed substantially between the pine and the ginkgo model (Fig 6B and 6E). For the pine, the ratio was about three whereas for the ginkgo it was about seven. For both models, the largest values occurred when the sonar was oriented 90 degrees to the side and the smallest values when the sonar was pointed directly at the model foliage. For the changes in beamwidth, all foliage models displayed a decrease in the values of the inhomogeneity measure with increasing beamwidth (Fig 6C and 6F). For the pine model, this decrease was drastic (by a factor of approximately 10) whereas for the ginkgo model it was only by a factor 2.

**Fig 7:**
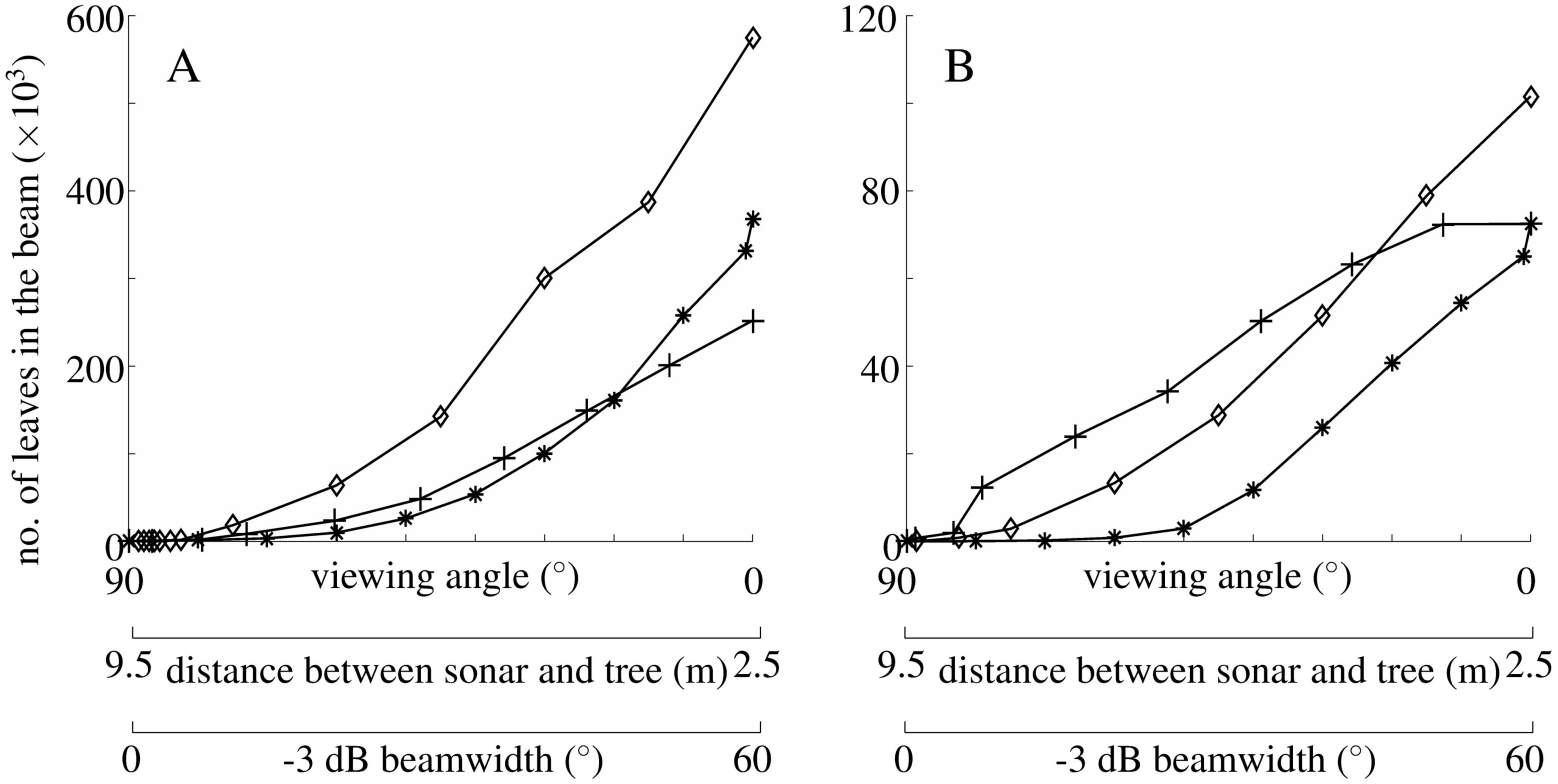
Relationship between the pose of the sonar beam and the number of leaves it contains. The number of leaves is given for the volume enclosed by the -80 dB gain surface of the biosonar beam: A) pine, B) ginkgo. Experimental paradigms (Fig 6): plus signs: approach; asterisks: rotational scan; diamonds: change in -3 dB beamwidth.

The direction in which the three experimental conditions impacted the inhomogeneity measure coincides with its impact on the number of leaves that were present in the biosonar beam (Fig 7). Whenever large numbers of leaves were in the biosonar beam, the inhomogeneity measure used here showed low values that decreased with an increasing number of leaves being contained in the beam.

## Discussion

The homogeneous spatial distribution of the leaves in the basic foliage model developed by the authors constitutes an obvious deviation from the situation in natural foliages where the distribution of the leaves is determined by a branching pattern. The results obtained here show a consistently larger temporal inhomogeneity in simulated echoes derived from models with biomimetic branching patterns than in echoes from models with a uniform spatial distribution of the leaves [11]. However, while highly consistent, these differences were also very small compared to the changes in temporal homogeneity seen as a result of varying the relative position of the sonar beam and the foliage. It remains a possibility that spatially inhomogeneous leaf distributions effect the echoes in ways that are not well represented by the temporal inhomogeneity measure that was introduced and evaluated here. However, if the distribution of the leaves varies along the propagation direction of the pulse, an effect on the echo properties over time seems to be a reasonable expectation. The two tree models that were tested here (ginkgo and pine) were very different in terms of their branching patterns and leaf densities, nevertheless they both have the same consistent results which could be taken as an indication of a general trend. If this is the case, it may not be necessary to sacrifice the simplicity of the uniform leaf distribution to get a reasonable fit to the behavior of natural foliages.

The relationship between temporal inhomogeneity in the echo amplitudes and the relative orientation of the sonar is a highly interesting feature found in the current simulated data set, in addition to the inhomogeneity resulted from leaf size and density [7]. This relationship may facilitate the bats’ sequential estimation [19] and also corresponds to how bats actually control their motions to classify the foliage information [20]. Since the temporal inhomogeneity was found to be a monotonic function of angle, distance to foliage, and beamwidth, it could be used by the bats to control these properties, e.g., during an angular scan, or derive an estimate of the relative orientation of the sonar beam that could be used as a prior knowledge in the estimation of other foliage properties. This could alleviate, for example, the problem that the authors previously encountered when trying to estimate all three parameters of the simplified foliage model (average leaf size, density, and orientation, [11]). If the bat was able to figure out the relative angular orientation of its sonar beam to the foliage surface from the temporal inhomogeneity of the echo envelope, this would allow the animals to predict the orientation of the leaves (assuming the leaf surfaces are oriented in parallel with the foliage surface), leaving only two parameters to be estimated from the echoes.

Differences in geometry (e.g., outline, folding) are a conspicuous feature of natural foliages that is not captured by the simplified numerical foliage model studied here. However, the experimental results obtained confirm that a simple disk geometry is able to mimic the overall trend in the relationship between leaf size and target strength. The steeper trend that the scattering strength of the 100 leaves follows might be accounted for by the lack of data in the range of [0, 1] cm and [2, 3] cm. The variability in the experimental results can be partially attributed to measurement errors (as evident from the repeated measurements undertaken) and partially to the geometrical complexity of the leaves that is not accounted for by the model. Leaf geometry can hence be seen as an additional factor that increases variability into the echo contributions from individual leaves. It may hence be possible to further improve the current model by using a different probability density function for the leaf size to incorporate this source of variability.

In the shading experiments, a “fully-shaded” disc sometimes returned a stronger echo than a “half-shaded” disc at the same distance. A pure shading mechanism would predict the opposite effect. It could be speculated that waves reaching the second disc on a direct path or after diffracting around the first disc interact in ways where in-phase additions results in higher amplitudes and out-of-phase additions in reduced amplitudes. Further experiments would be needed to establish the mechanisms, but for the purpose of the present study it is sufficient to note that these interactions exist.

The interaction effects found in the experiments between the leaves of a foliage could be an important factor in determining the contribution of leaves inside the foliage on the echoes. Leaves in many natural foliage types are frequently spaced less than 25 cm apart and hence could produce substantial shading. It has been reported that echoes from dense and sparse foliages differ in the attenuation of echo amplitudes over time [3]. But in our current model, shading was not considered, indicating that echoes from sparse and dense vegetation were calculated in the same way. Hence, the current model may become more accurate with an adjusted attenuation function to incorporate the effect of shading between leaves and sonar pulse, especially for dense foliage. The ideal solution to both issues with the current model (variable leaf geometry and shading) would be based on further insights into how the physical mechanisms can be represented accurately with a mathematical model but in a much simpler manner than simulating diffraction by arbitrary leaf geometries and taking into a account the relative positions of the leaves. Future research is needed to establish whether this is possible.

In summary, the physical and modeling experiments carried out here demonstrate that the simple three-parameter model is well suited to approximate natural foliage echoes. Future research should target improvements that can be made to the model (e.g., addition of a shading function) as well as explore new hypotheses for how the properties of the echoes can be used to support sonar-based navigation in natural environments.
